# Forest loss in New England: A projection of recent trends

**DOI:** 10.1371/journal.pone.0189636

**Published:** 2017-12-14

**Authors:** Jonathan R. Thompson, Joshua S. Plisinski, Pontus Olofsson, Christopher E. Holden, Matthew J. Duveneck

**Affiliations:** 1 Harvard Forest, Harvard University, Petersham, MA, United States of America; 2 Dept. of Earth & Environment, Boston University, Boston, MA, United States of America; Clemson University, UNITED STATES

## Abstract

New England has lost more than 350,000 ha of forest cover since 1985, marking a reversal of a two-hundred-year trend of forest expansion. We a cellular land-cover change model to project a continuation of recent trends (1990–2010) in forest loss across six New England states from 2010 to 2060. Recent trends were estimated using a continuous change detection algorithm applied to twenty years of Landsat images. We addressed three questions: (1) What would be the consequences of a continuation of the recent trends in terms of changes to New England's forest cover mosaic? (2) What social and biophysical attributes are most strongly associated with recent trends in forest loss, and how do these vary geographically? (3) How sensitive are projections of forest loss to the reference period—i.e. how do projections based on the period spanning 1990-to-2000 differ from 2000-to-2010, or from the full period, 1990-to-2010? Over the full reference period, 8201 ha yr-1 and 468 ha yr-1 of forest were lost to low- and high-density development, respectively. Forest loss was concentrated in suburban areas, particularly near Boston. Of the variables considered, 'distance to developed land' was the strongest predictor of forest loss. The next most important predictor varied geographically: 'distance to roads' ranked second in the more developed regions in the south and 'population density' ranked second in the less developed north. The importance and geographical variation in predictor variables were relatively stable between reference periods. In contrast, there was 55% more forest loss during the 1990-to-2000 reference period compared to the 2000-to-2010 period, highlighting the importance of understanding the variation in reference periods when projecting land cover change. The projection of recent trends is an important baseline scenario with implications for the management of forest ecosystems and the services they provide.

## Introduction

Forest conversion to developed uses is a significant and pervasive agent of global change [1]. Worldwide, land clearing for human settlements are expanding [2,3] and nearly 20 percent of global forests are now within 100 meters of a non-forest edge [4]. In the United States (U.S.), developed land is the most rapidly expanding land cover class while forest land is the most rapidly declining [5]. Forest loss and fragmentation are primary causes of habitat loss and associated declines in biodiversity [6,7]. In addition, conversion of forests to developed uses has significant consequences in terms of ecosystem service provisioning, including: regulating services such as carbon storage and flood attenuation, provisioning services such as timber and wild food production, and cultural services such as outdoor recreation. A better understanding of the patterns, drivers, and trends associated with forest loss is a critical frontier in sustainability science [8,9].

In the northeastern U.S. a one hundred fifty-year-old trend of forest expansion that took the region from approximately 40% to 80% forest cover has recently reversed and the region is again losing forest-cover [10–13]. Concern for the fate of the region’s forests and its natural infrastructure have led to calls for broad-scale land protection [e.g., 14,15] and spurred scenario studies to help understand and anticipate alternative future land-use trajectories [e.g., 16,17]. Here we examine the rates and distribution of forest loss in the northeast U.S. and quantify implications of a potential continuation of this second wave of regional-scale forest loss.

Land change models (LCM) are valuable tools for developing a landscape-specific understanding of past and potential future changes to landscapes, including forest loss. These models take a variety of forms—from process-based to phenomenological—each with advantages and disadvantages that are contingent on the application (see [18] for a review of LCMs). Irrespective of the land use change in question, a common first step when using LCMs is to project a linear continuation of the recent trends in terms of the rate and spatial pattern observed in land change transitions—i.e. a business-as-usual scenario. Such projections are often interpreted as predictions, but given the high uncertainty and low predictive power of LCMs [19–21], they are better used as a baseline or benchmark for evaluating a broad suite of land-change scenarios. In this regard, projections of recent trends can serve as useful scenarios of future land change that can be compared with alternative scenarios.

Cellular LCMs are often used for projecting a continuation of observed recent trends of land-cover change [18], and thus operate with the implicit assumptions that the future will be an undeviating continuation of the past. These models quantify statistical relationships between observed patterns of land-cover change (typically derived from remote sensing) within discrete spatial units (cells) within a landscape and include ancillary social and biophysical features. These relationships are then used to project change into the future. The reference period used, therefore, determines the attributes of the future projections. Cellular LCMs are frequently trained with land cover maps derived from remote sensing and the reference period have often been dictated by the availability of adequate land cover maps [20]. However, increasing availability of remote sensing data (e.g. making Landsat data archive free of charge [22] along with advances in algorithms to map and monitor land cover have culminated in continuous time series of land cover change with relatively high spatial detail [23,24]. This presents an opportunity to compare patterns of forest loss and fragmentation using multiple recent trends projections that are based on alternative reference periods.

Implicit within the use of cellular LCMs is that relationships between land change and the predictor variables that determine suitability for change are stationary throughout the study area. These relationships are often well established at local scales. For example, the relationships between infrastructure and land cover change, including the distance from roads [3,25–31], the distance to urban centers [12,30,32], and distance to previously developed areas [28,30,33,34] have been associated with the probability of forest conversion. Similarly, physical attributes such as slope [3,33,35], and wetlands (which can have regulatory and biophysical impacts on development) [36], and social attributes, including population density [30,32,37,38] and ownership [39] have all been used to explain variation in local patterns of land change and to project change into the future. However, caution is warranted when projecting change over large areas where the nature of these relationships may vary widely. By fitting models independently to sub-regions where similar patterns of land use change exist within a larger study area, cellular LCMs can allow for the variation in the strength and form of these relationships [40].

In this study we used Dinamica EGO, a cellular LCM, to project the recent trends of forest loss within 32 sub-regions and three temporal reference periods within northeastern U.S. We address three specific research questions: (1) What are the potential consequences to New England’s forest cover (in terms of forest area and fragmentation) if recent trends of forest loss continue for fifty years? (2) What social and biophysical attributes are most strongly associated with the spatial patterns of forest loss and how do these vary across the region? (3) How sensitive are projections of forest cover loss to the reference period used to build the model—i.e. how do projections based on the period spanning 1990 to 2000 differ from 2000 to 2010, or from 1990 to 2010?

## Methods

**Fig 1:**
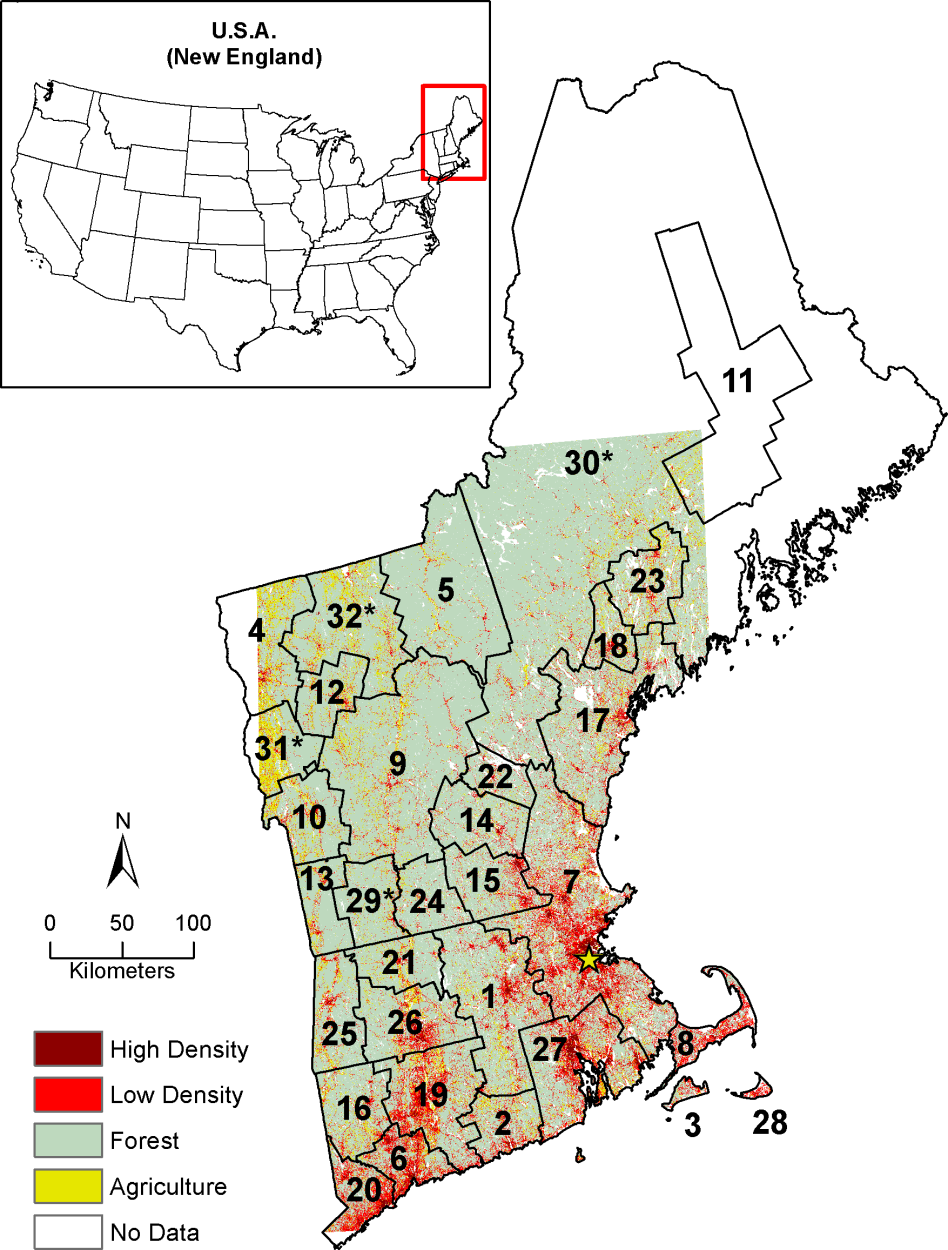
Study area showing the 32 sub-regions used in the Dinamica simulations. These sub-regions include 27 Core Base Statistical Areas (CBSA) as defined by the U.S. Census and 5 non-CBSA regions. Non-CBSA regions are denoted with an asterisk. Additionally, the three sub-regions chosen as case studies are Boston-Cambridge-Newton (7), Claremont-Lebanon (9), and Portland-South Portland (17).

Our 112,000 km^2^ study area in the northeastern U.S. includes all of Massachusetts and New Hampshire, 93% of Vermont, 99% of Connecticut, and approximately 33% of Maine (Fig 1). This area was delineated based on the footprint of six Landsat scenes and was described by [13], who applied the Continuous Change Detection and Classification (CCDC) algorithm to a pixel-level time-series of 30m Landsat data [13,24]. The CCDC algorithm utilizes all available Landsat data to identify multiple types of land cover change for a given time period. The CCDC classification of New England included 13 classes of land cover [13]. For this study, we used land-cover maps from 1990, 2000, and 2010 and reclassified the data into: (1) a single forest class, (2) high density development, (3) low density development, and (4) an “all other” class (see reclassification table in S1 Table). The high density development class encompasses areas of urban or residential development with impervious surface areas from 50% to 100%. The low density development class encompasses areas of urban or residential development with impervious surface areas from 0% to 50%.

To account for regional variation in the patterns and drivers of land cover change, we delineated 32 sub-regions within the study area, within which, we independently fit models of land-cover change. The sub-regions are based on U.S. Census Bureau defined Core Base Statistical Areas (CBSA) which collectively represent both Census Metropolitan and Micropolitan statistical areas (www.census.gov; accessed 4/20/2017). CBSAs are delineated to include a core area containing a substantial population nucleus, together with adjacent communities having a high degree of economic and social integration with that core. New England includes 27 CBSAs, however not all of New England is covered by a CBSA. Accordingly, we added five rural regions to fill the gaps, for a total of 32 unique sub-regions (Fig 1). We excluded legally protected forest from future development using the Conservation Biology Institute Protected Areas Database [41].

Within each sub-region and time-period, we summarized the mapped rate of forest lost to development. We adjusted these mapped rates using the ‘good practices’ methods outlined by [42] to account for the bias identified in the accuracy assessment of the classification. The satellite-based analysis of Olofsson et al. [13] omitted a significant portion of forest converted to low density development, usually along the edges of developments or when conversion took place but retained significant forest cover. They quantified the extent of the omission based on a sample of points manually interpreted from aerial imagery, from which they developed bias estimates. We incorporated these bias estimates to set the rate of forest loss. The bias adjustment multiplier for the forest-to-low-density-development category was 3.61. The bias in the mapped area of forest to high density development was 0.9. We applied these adjustments to the projected rates of conversion uniformly across the sub-regions, as there was no spatial bias identified in the accuracy assessment [13]. The accuracy assessment did not attempt to quantify potential biases in the other aspects of the classification, such as temporal trends or patch size or shape. Therefore, we used their unadjusted estimates within our simulation parameterization.

We used Dinamica Environment for Geoprocessing Objects (Dinamica EGO 2.4.1) to project fifty years (2010 to 2060) of future forest loss under a “Recent Trends” scenario using five ten-year time steps. Dinamica EGO is a spatially explicit cellular automata model of landscape dynamics capable of multi-scale stochastic simulations that incorporate spatial feedback [43]. Dinamica EGO is used globally to simulate land cover change [44–46]. Within Dinamica EGO there are five parameters that most influence the patterns of land cover change and that we estimated from the reference maps: the transition rate, the ratio of new vs. expansion patches, the mean and variance of new patch sizes, and patch shape complexity (i.e., patch aggregation). Our procedure for estimating these parameters was as follows: (1) The estimated forest loss rate for each sub-region was defined as the mapped area of forest that transitioned to high- and low-density development within each region and reference period, adjusted by the bias adjustment modifier as described above. (2) The ratio of new patches to expanding patches was calculated from the ratio observed in the reference periods within each sub-region. (3) The quantity and size of newly generated patches was based on a normal distribution controlled by the mean patch size and variance observed in each reference period. Dinamica generates new patches by; first selecting a "seed cell" from a set of candidate cells, the patch then expands iteratively into neighboring pixels with the highest transition probability until it met its size quota. (4) Patch shape complexity is controlled by an isometry parameter, which is a multiplier that increases or decreases the underlying transition probability values of neighboring cells around a seed cell. As a patch expands from its initial seed cell, a 3x3 moving window is placed over every patch cell. Within in this 3x3 window, the underlying probability map is multiplied by the isometry value. Values greater than 1 will increase the likelihood that patches will be simpler, more aggregated shapes. Values less than 1 will result in more complex shapes (i.e, less aggregated). We found the isometry value of 1.1 best matched the patch shape complexity observed in the reference period transition patches for the transitions from forest to development.

We examined patterns of forest loss within each sub-region in relation to a suite of spatial predictor variables (Table 1). We selected variables that have been shown to be associated with rates and patterns of forest loss (as reviewed in the *Introduction*) and that were not inter-correlated (i.e. Pearson’s < |0.7|, sensu [47] (Table 1)). Dinamica EGO employs a weights-of-evidence (WoE) approach to set the transition probability for any given pixel. The WoE method uses a modified form of Bayes theorem of conditional probability [48,49] to derive weights where the effect of each spatial variable on a transition is calculated independently of a combined solution [50]. The method requires that continuous variables be discretized through an iterative binning process so that individual weights can be calculated for each bin. We modified the algorithm to only create a new bin if the difference would result in a statistically significant difference in the probability of transition between bins. Dinamica calculates separate weights (W+) for each driver variable independently then sums the W+ values to create the composite transition potential map. For each driver variable, positive W+ values predict the future occurrence of new development patches while negative W+ values predict the future absence of new development patches. The highest W+ values in the composite transition map represent the sites with greatest potential for transition using the combined predictive power of all driver variables. Stochasticity within the patch selection process is controlled by thresholding the transition potential map to create a subset of high W+ candidate cells for new patch seeding. We used the default setting within Dinamica EGO, which multiples the number of expected transition cells for each transition by 10 to create a pool of candidate cells, from which seed cells are selected randomly for transition. Once the seed cell is selected, patch formation itself is not stochastic. The patch will iteratively expand into neighboring cells based on the underlying probability values until the patch size quota is met.

**Table 1:** Variables used to predict spatial location of forest loss within Dinamica EGO land cover simulations.

Variable	Units	Minimum Bin Size	Source
Distance to Development	Meters	100 m	Olofsson et al. 2016
Distance to Urban Areas (*included cities within 100km buffer to study area boundary).	Meters	10,000 m	U.S. Department of the Census 1990, 2010.
Distance to Roads/Highways	Meters	100 m	U.S. Department of the Census 1990, 2010.
Slope	Degrees	2°	United States Geological Service 2016
Land Owner Type	Categorical	NA	Sewall GIS Services 2015 http://www.sewall.com/services/geospatial/gis.php
Wetlands	Categorical	NA	U.S. Fish and Wildlife Service 2016, Federal Emergency Management Agency 2016, United States Geological Service 2016.
Population Density	People/Square Kilometer	25 ppl / sq. km.	U.S. Department of the Census 1990, 2010.

To evaluate the effect of reference time period used, we simulated 50 years of land cover change using three separate reference periods: 1990 to 2000, 2000 to 2010, and 1990 to 2010. The same predictor variables were used in each simulation and all simulations were run from 2010 to 2060. We evaluated the impacts of land cover change in terms of changing forest cover and edge density for the full study region and within sub-regions using the Raster [51] and SDM [52] libraries within the R statistical software [53]. We elected to use edge density to summarize changes in forest fragmentation because it is both intuitive (it quantifies the meters of forest/non-forest edge in proportion to total forest area) and because it is relatively insensitive to the total amount of forest at intermediate levels (i.e. when forest area is >30% and < 70% of the forest area) [54].

To evaluate the relative importance of individual predictor variables by sub-region, we compared the mean W+ values for each independent driver variable at the location of newly developed patches during the first time-step. We also selected three example regions, Boston-Cambridge-Newton, Claremont-Lebanon, and, Portland-South Portland to highlight how W+ values of individual predictors varied across their range and between these sub-regions. We selected these examples to showcase three qualitatively different types of CBSA with distinct patterns of forest loss. Boston-Cambridge-Newton CBSA contains a high density metropolitan area surrounded by established satellite cities and suburbs and has a high rates of forest loss and the greatest constraints on where forests can be developed. Portland-South Portland CBSA is medium sized city surrounded by a mix of suburbs, forests patches, and agricultural land that is developing at a moderate rate. Claremont-Lebanon is a rural CBSA where the rate of development much slower with multiple small urban centers surrounded by large areas of core forest, rugged mountains, and agricultural valleys Finally, we compared the impacts of the three temporal reference periods in terms of differences between Year-2060 forest area, edge density, and the importance of predictor variables within the simulations.

## Results

### Projections of forest cover change based on the full 20-year (1990–2010) reference period

**Fig 2:**
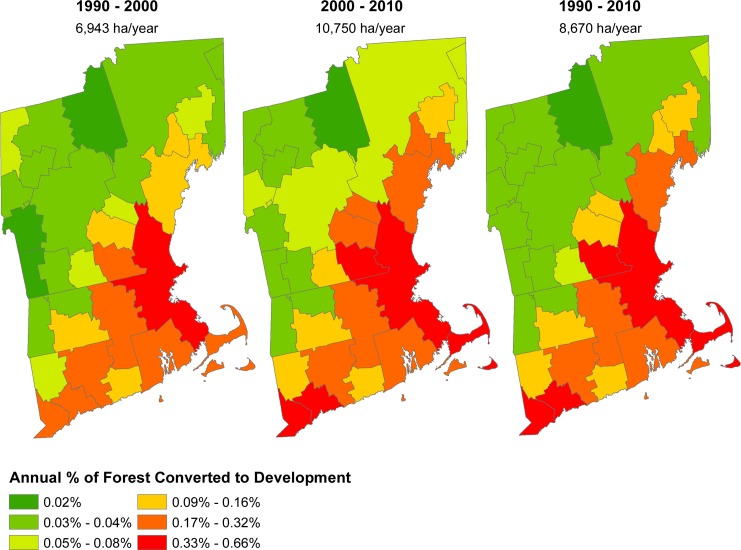
Rates of forest conversion to development for the three reference periods used in this study: 1990–2000, 2000–2010, and 1990–2010. Value below each reference period title signifies total forest area lost per year.

The rate of forest loss to low-density development during the full 20-year reference period (1990 to 2010) was 8,201 ha yr^-1^, or 0.1% yr^-1^. Forest lost to high density occurred at a rate of 468 ha yr^-1^ or 0.006% yr^-1^. During the 50-year simulations, 433,500 ha of forests were converted to developed uses throughout the study area, resulting in a decline in forest cover from 75.2% in 2010 to 71.2% in 2060 (Fig 2). Twenty-two percent of total forest loss occurred within the Boston-Cambridge-Newton region, even though it contained just 5.8% of the region’s forest in 2010. Forest cover declined from 51.8% to 41.2% in this sub-region. Across the landscape, forest loss was concentrated in the southern sub-regions, which either contain or are near large urban centers, including the cities of Hartford, CT and Providence, RI. In contrast, the sub-regions without large urban hubs experienced little forest loss. Indeed, the 21 regions with the most stable forest cover (40% of the total area) lost less forest cover than did Boston-Cambridge-Newton (8% of total area).

**Fig 3:**
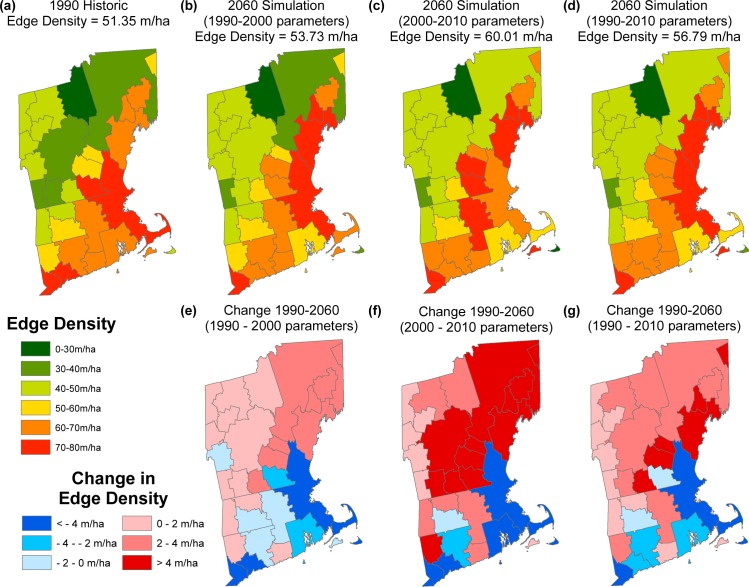
Edge density by sub-region (a, b, c, d) and total edge density (text above each map) for 1990 (a), and 2060 using three reference periods (b, c, d). Change in edge density by sub-region using three reference periods and fifty years of simulation (e, f, g).

Simulated changes in edge density (our measure of forest fragmentation) at the region scale contrasted with the patterns of forest loss. Edge density declined in the more developed sub-regions where forest loss was greatest, (e.g., Boston-Cambridge-Newton) (Fig 3). Decreasing edge density is the result of in-filling of the developed classes and the loss of small, edgy forest patches. Sub-regions adjacent to the more developed sub-regions (e.g., first-order neighbors to Boston-Newton-Cambridge region) experienced the largest increases in edge density as forest cover became more perforated and the land cover mosaic more complex. This included the 11.75% increase (+6.87 m/ha) in Concord, NH (sub-region #14) and 11.10% increase (+6.24 m/ha) in Laconia, NH (sub-region #22), which experienced the highest increases of all sub-regions, respectively. After 50-years of simulated forest loss (2010–2060), regional-scale edge density increased by 10.59% from 51.35 m/ha to +56.79 m/ha and change in edge density by sub-region ranged from an increase of 11.75% (+6.87 m/ha) to a decrease of 16.92% (-12.02 m/ha).

### Landscape attributes associated with patterns of forest loss (based on the full 20-year reference period)

**Fig 4:**
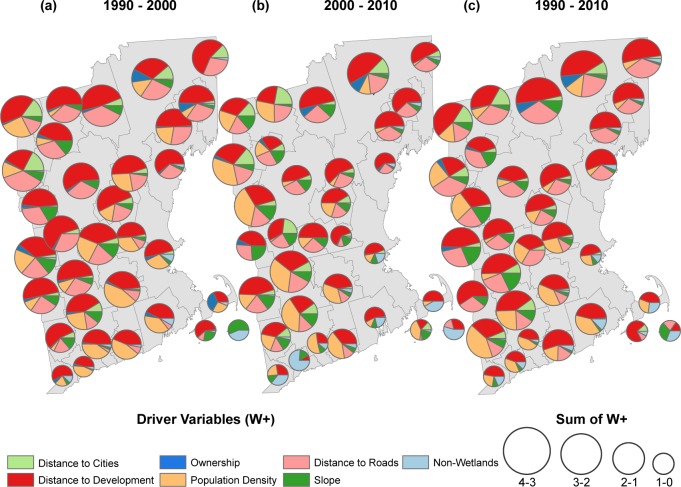
Forest to low density development weights. Mean positive W+ by sub-region for simulated transitions from forest to low density development during the first time-step of simulation. Size of pie charts is proportional to the mean sum of W+. Proportions within pie charts show relative contribution of W+ within sub-regions.

**Fig 5:**
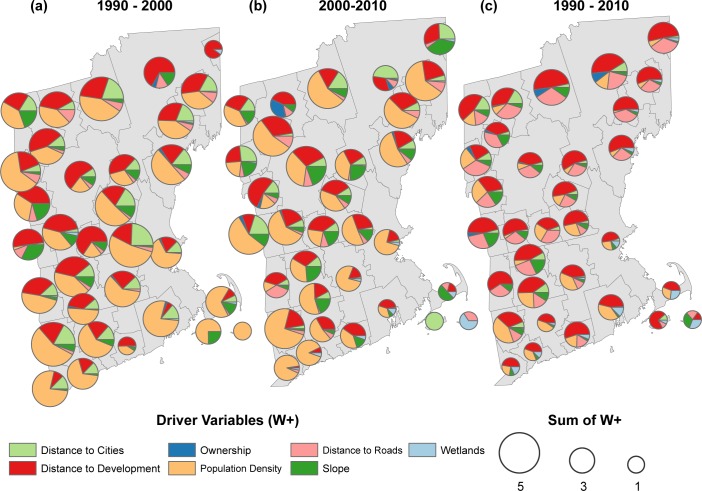
Forest to high density development weights. Mean positive W+ by sub-region for simulated transitions from forest to high density development during the first time step of simulation. Size of pie charts is proportional to the mean sum of W+. Proportions within pie charts show relative contribution of W+ within sub-regions.

Of the variables considered, ‘distance to the nearest developed land’ was the strongest predictor of forest loss to low density development in 29 out of 32 sub-regions (Fig 4). The next most important predictor for this transition varied between ‘distance to roads’ (16 out of 32) and ‘population density’ (11 out of 32). There were clear geographical differences in the secondary predictors. In the more developed (and developing) southern regions, population density was more likely to be the second most important driver, whereas in the less developed northern regions distance from the nearest road was more often to be the second most important driver. Along the coast, wetlands had a strong negative associated with forest conversion. Slope had a strong negative association in the mountainous northern and eastern regions. Forest conversion to high-density development was a much less frequent transition than to low-density development, so the variables had lower W+ and were less predictive. But, in general, high-density development was positively associated with population density (Fig 5).

**Fig 6:**
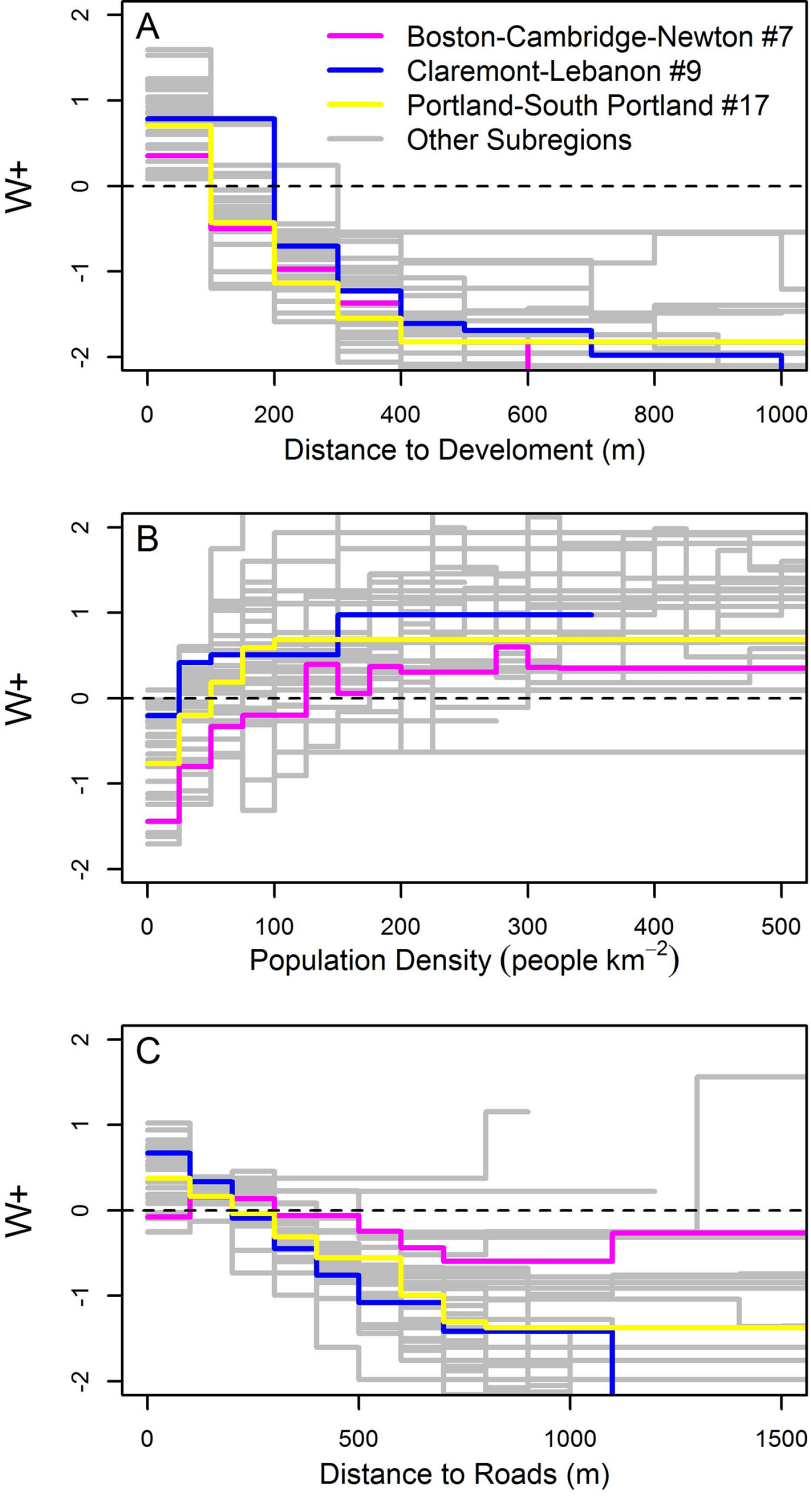
**Variation in W+ for distance to development (A), population density (B), and distance to roads (C) for three example sub-regions.** See Fig 1 for numbered sub-region locations. Weights (W+) above zero increase the probability of development; weights below zero decrease probability of development.

Beyond the rank order of their importance, the effects of the spatial predictors on the patterns of forest conversion tended to follow similar patterns but the magnitude of the W+ varied among the 32sub-regions (Fig 6). For example, W+ values decreased with increasing distance to the nearest developed area in every CBSA. In the rural Claremont-Lebanon sub-region in New Hampshire, distance to the nearest developed area had a positive influence on the probability of low density development within a 200m radius, beyond which the distance to development had a negative influence. In contrast, in the more developed Boston-Cambridge-Newton and Portland-South Portland sub-regions, the distance to development was positive within a 100m radius and then turned negative (Fig 6A). Even small differences in the point at which W+ transitions from positive to negative, such as between 100 and 200m to the nearest development, results a distinct patterns forest loss within regions with high development density. Similarly, population density in Claremont-Lebanon had a positive influence above 25 people km^2^; but in Boston-Cambridge-Newton, population density did not increase the probability of conversion until population was greater than 125 people km^2^ (Fig 6B). In general, development was also associated with short distances to roads (Fig 6C). In rural sub-regions, the W+ was positive for short distances. In Boston-Cambridge-Newton, however, distances less than 100m from a road had a slight negative W+ where what little forest exists was unsuitable for development. In general, greater distances from roads were associated with negative W+ and decreased the farther a forest pixel occurred from a road. Compared to the rural regions, the magnitude of W+ was lower in the Boston-Cambridge-Newton sub-region (Fig 6C). Such differences underscore the importance, as we have done, of fitting independent models to socially and economically distinct sub-regions within the study area.

### The impact of the reference period on projected forest conversion

Forest-loss in the 1990 to 2000 reference period occurred at a rate of 6943 ha yr^-1^, 35% lower than during the 2000 to 2010 period when forest loss occurred at 10750 ha yr^-1^. When using the full 1990 to 2010 reference period, the projections resulted in 8670 ha yr^-1^ of forest loss (approximately the average of the earlier and later periods). The ratio of high to low-density development was relatively consistent between the early and later reference period: 1: 19.2 and 1: 17.9, respectively. While, the choice of reference period resulted in a difference of as much as 190,000 hectares of forest loss to development from 2010 to 2060, the spatial patterns of forest loss were consistent over time. Indeed, the same six sub-regions had the highest rate of forest loss in both 10-year reference periods. The Boston-Cambridge-Newton region contained 24% of the region’s forest loss in the 1990–2000 period and 20% in the 2000 to 2010 period.

With some notable exceptions, the relative importance of the available social and biophysical predictor variables varied across the study area but was consistent over the time periods. Distance to development was the most important predictor of forest loss to low-density development in each time period. The shorter 10-year reference periods had less land cover conversion that was used in the model fitting. Therefore, the relative weights of evidence for the predictor variables were more variable as the result of fewer transitions.

## Discussion

We projected the recent trends in forest loss within New England and evaluated potential consequences to the regional landscape. Rates and patterns of projected forest loss were highly variable across the region. Forest loss occurred overwhelmingly in suburban environments, especially in the Boston-Cambridge-Newton sub-region where forest cover declined to 41%, by far the lowest in the region. This pattern of forest loss sprawling out from urban centers has been observed many times over in the northeast U.S. [55,56]. And, globally, proximity to cities correlates with greater rates of land cover change, specifically with the loss of forest and farms [57,58]—this pattern is at the heart of the longstanding notion of ‘Central Place Theory’ [59]. Given this, projections of continued suburbanization may help planners and policy makers anticipate coming changes and protect the remaining forests fragments, which are often hotspots of ecosystem service provisioning [60].

While sub-regions with large urban centers lost the most forest cover, it was the adjacent sub-regions that experienced the greatest increases in edge density. In fact, edge density declined in the urban sub-regions as new developed areas in-filled the already fragmented landscape. The bordering sub-regions experienced increases in edge density of as much as 11.75% (+6.87 m/ha) under the 1990–2010 reference period and 22.42% (+13.1 m/ha) for the 2000–2010 reference period, despite comparatively low losses in forest area. This finding underscores the importance of total forest area as well as configuration. These areas in the outskirts of Boston have been referred to as the “sprawl frontier” (sensu, [61]). These areas currently remain dominated by forest cover but were shown to be vulnerable to fragmentation within these projections.

Our second question concerned the regional variation in amount of forest loss and the variation in the strength of the predictor variables used to project new development. We effectively captured the variation in the development patterns at both the local within-region scale, and the regional between-region scale. For example, across the study area, population density ranged from a very important predictor of low density development in many southern regions, explaining as much as 50% of the total positive weight of evidence for simulated transitions, to unimportant (< 15%) in the some of the northern regions (Fig 3). Then, looking within regions, weights associated with population density varied between regions. As noted above, in Boston-Cambridge-Newton (sub-region #7) the weight associated with population density was negative below ~150 people km^-2^, while in Clarmont-Lebenon (sub-region #9) population density greater than 25 people km^-2^ had positive weight (Fig 6B). Had we taken the more conventional approach of developing a recent trends simulation for the whole region, the magnitude of forest loss near the urban areas (such as in Boston-Cambridge–Newton) would have overwhelmed the patterns of forest loss, and rural areas may not have ever accumulated the weight to result in a land cover transition. By simulating the transition rates and the driver variable weights within sub-regions separately, we were able to preserve the regional spatial patterns of transitions and capture the varying influence of different predictors across the study area.

Our final research question examined the impact of the reference period selected to project forward in a recent trends simulation. We compared two ten-year intervals (1990–2000 and 2000 to 2010) and the encompassing 20-year period (1990 to 2010). There was a 55% more forest loss in the 1990–2000 compared to the more recent 2000–2010 reference period but strong consistency in the broad regional-scale spatial patterns, with a near-perfect correlation in the rank order of forest loss by sub-region. The low number of transitions within some regions during the ten-year periods resulted in some volatility in terms of predictor importance. For example, the owner-class variable (i.e. public or private) had a relatively high weight within the Barnstable Town, MA in 1990 to 2010 but had no predictive weight in the 2000 to 2010 period. Further investigation showed that, during the first time-step, there was little land cover change within a large federally owned military base, which resulted in a significant difference between public and private land. In 2010, a conservation restriction was put on the military base, which made the land ineligible for conversion, per our methodology. Without that block of public land in contention for development, the importance of owner-class was insignificant. Such volatility in the relative importance of predictors, underscores the value of longer reference periods and an understanding of regional land-use dynamics, especially where there is less land cover change used to fit statistical models. Fortunately, greater availability of remote sensing data (e.g. the opening of the Landsat archive) coupled with advances in land cover change mapping (e.g. [23,24] are resulting in long time series to train cellular land cover change models.

## Future directions

These projections of recent trends in land cover change are not predictions. They represent one useful scenario that can help us understand some the current landscape land cover dynamics and the potential consequences of the current trajectory. Indeed, we developed these projections as part of the New England Landscape Futures project, which is an initiative to develop a suite of land-use scenarios with the dual objectives of advancing research and informing sustainable land-use policy and planning in New England [62]. Within the context of the project we have used the recent trends projections to inform alternative future scenarios. These projections and associated analyses were shown to groups of stakeholders attending workshops throughout region that were held as part of a participatory scenario development process. Information from the recent trends projections served as their common base of information as they crafted a suite of alternative land-use scenarios currently in development. The recent trends simulations served as starting point for the simulations of the alternative scenarios. The Dinamica EGO model and the Weights of Evidence approach it employs are well designed for this purpose. We use the information given in the stakeholder’s scenarios into new land cover transition rates as a baseline and we adjust the weights associated with predictor variables (sensu, [63]. Ongoing research couples these recent trends projections with spatial simulations of forest growth, climate change, and land use such as timber harvesting. Together the model projections will allow an analysis of the relative effects of land use on future landscape carbon sequestration, and other critical forest-based ecosystem services.

## Supporting information

S1 Table Land cover reclassification table. Continuous change detection land cover classes and their reclassified land cover classes used in this study.
(PDF)
