# Supplementary material for: Forest loss in New England: A projection of recent trends
Source: PLoS One. 2017 Dec 14;12(12):e0189636. doi: 10.1371/journal.pone.0189636 (PMC5730125; doi:10.1371/journal.pone.0189636)
Supplement: S1 Table — Continuous change detection land cover classes and their reclassified land cover classes used in this study. (PDF) [file pone.0189636.s001.pdf]

CCDC Class	CCDC Description	Reclassification
Disturbed	Areas of no-data or missing data due to clouds, shadows, etc.	No Data
Bare	Non-vegetated land comprised of above 60% rock, sand, or soil	Other
Commercial/Industrial	Area of urban development; impervious surface area target 80-100%	High Density Developed
High Density Residential	Area of residential urban development with some vegetation; impervious surface area target 50-80%	High Density Developed
Low Density Residential	Area of residential urban development with significant vegetation; impervious surface area target 0-50%	Low Density Developed
Herbaceous / Grassland	Non-woody naturally occurring or slightly managed plants; includes pastures	Other
Agriculture	Non-woody cultivated plants; includes cereal and broadleaf crops	Agriculture
Mixed Forest	Forested land with at least 40% tree canopy cover comprising no more than 80% of either evergreen needleleaf or deciduous broadleaf cover	Forest
Deciduous Broadleaf Forest	Forested land with at least 40% tree canopy cover comprising more than 80% deciduous broadleaf cover	Forest
Evergreen Needleleaf Forest	Forested land with at least 40% tree canopy cover comprising more than 80% evergreen needleleaf cover	Forest
Wetland	Vegetated land (woody and non-woody) with inundation from high water table; includes swamps, salt and freshwater marshes and tidal rivers/mudflats	Other
Water	Lakes, ponds, rivers, and ocean	Other
Woody Wetland	Additional class of wetland that tries to separate wetlands with considerable biomass from mainly herbaceous wetlands	Forest
NoData	NoData	No Data
